# A metabolomics study of Qiliqiangxin in a rat model of heart failure: a reverse pharmacology approach

**DOI:** 10.1038/s41598-018-22074-6

**Published:** 2018-02-27

**Authors:** Junzeng Fu, Liping Chang, Amy C. Harms, Zhenhua Jia, Hongtao Wang, Cong Wei, Li Qiao, Shuyan Tian, Thomas Hankemeier, Yiling Wu, Mei Wang

**Affiliations:** 1 Department of Analytical Biosciences, Leiden Academic Center for Drug Research, Leiden University, Einsteinweg 55, 2333 CC Leiden, The Netherlands; 2 Hebei Medical University, Shijiazhuang, Hebei, 050017 P.R. China; 3 Yiling Hospital of Hebei Medical University, The Key Laboratory of State Administration of Traditional Chinese Medicine, Shijiazhuang, Hebei, 050091 P.R. China; 4 Netherlands Metabolomics Centre, Leiden University, Einsteinweg 55, 2333 CC Leiden, The Netherlands; 5 National Key Laboratory of Collateral Disease Research and Innovative Chinese Medicine, Shijiazhuang, Hebei, 050035 P.R. China; 6 Hebei Key Laboratory of Collateral Disease, Shijiazhuang, Hebei, 050035 P.R. China; 7 LU-European Center for Chinese Medicine and Natural Compounds, Institute of Biology, Leiden University, Sylviusweg72, 2333BE Leiden, The Netherlands; 8 SU BioMedicine, Sylviusweg 72, 2333BE Leiden, The Netherlands

## Abstract

The Chinese medicine Qiliqiangxin (QL) has been shown to have a protective role in heart failure. Here, we explore the underlying working mechanism of the key therapeutic component in QL using a rat model of heart failure. Heart failure after myocardial infarction was induced surgically and confirmed using echocardiography; a separate group of rats underwent sham surgery. The rats with heart failure were randomly assigned to receive QL, the angiotensin-converting enzyme inhibitor benazepril, or placebo groups. Blood samples were collected from the rats at four time points for up to 8 weeks and used for biochemical analysis and mass spectrometry‒based metabolomics profiling. In total, we measured nine well-known biochemical parameters of heart failure and 147 metabolites. In the rats with heart failure, QL significantly improved these biochemical parameters and metabolomics profiles, significantly increasing the cardioprotective parameter angiopoietin-like 4 and significantly lowering inflammation-related oxylipins and lysophosphatidic acids compared to benazepril. Mechanistically, QL may improve outcome in heart failure by controlling inflammatory process and cardiac hypertrophy. Clinical studies should be designed in order to investigate these putative mechanisms in patients.

## Introduction

Heart failure is a complicated clinical syndrome that can result from any functional and/or structural cardiac disorder that impairs the ventricle’s ability to fill with or eject blood^1^. Heart failure is currently one of the leading causes of morbidity and mortality worldwide, placing a significant burden on patients, their families, and society^2^.

In recent decades, Western pharmaceutical-based therapies have shown promising effects in terms of improving symptoms and reducing heart failure‒related mortality by targeting pathogenic pathways; these therapies include angiotensin-converting enzyme (ACE) inhibitors, beta-blockers, and aldosterone antagonists^3^. However, these treatments are often associated with side effects, including electrolyte imbalance, fluid depletion, and hypotension^4^. In contrast to the reductionist, molecular target approach of Western medicine, Chinese medicine uses a holistic and personalized approach to both diagnosis and intervention of disease, as well as supporting health, with fewer side effects and lower costs compared to Western medicine^4^. Recently, using a systems approach to bridge Western and Chinese medicine at the biochemical level has yielded insight into the mechanisms that underlie the beneficial effects of Chinese medicines^5–7^, suggesting that Chinese medicine may provide a complementary therapy in patients with heart failure.

Qiliqiangxin (QL) is a well-known herbal medicine derived from a classic traditional Chinese formula. QL capsules contain herbal extract from the following herbs: *ginseng radix et rhizoma*, *astragali radix*, *aconm lateralis radix praeparaia*, *descurainiae semen lepidii semen*, *salviae miltiorrhizae radix et rhizoma*, *alismatis rhizoma*, *cinnamomi ramulus*, *polygonati odorati rhizoma*, *periplocae cortex*, *carthami flos*, and *citri reticulatae pericarpium*^8^. In China, QL has been used in clinical practice for treating heart failure for more than a decade. In addition, a multicenter randomized double-blind study showed that QL has cardioprotective benefits in patients with heart failure^9^. Finally, QL has been reported to reduce cardiac remodeling following acute myocardial infarction in mice^8^. Although these previous studies demonstrated the beneficial effects of QL, the underlying therapeutic effects and the underlying mechanisms in the context of heart failure remain poorly understood.

Heart failure is associated with a complex range of pathologies, including profound changes in cardiac metabolism^10^. Thus, several products of multiple cardiac metabolic pathways, including metabolites related to inflammation and energy metabolism, are important intermediates in the pathogenesis of heart failure^10–12^. Here, we used biochemical assays to measure several parameters that are commonly used as markers in heart failure‒related research. In addition, we used mass spectrometry‒based metabolomics profiling to measure a wide range of metabolites, including oxylipins, oxidative and nitrosative stress markers, and organic acids. Finally, we used these biochemical parameters and metabolomics profiles to systematically examine the molecular mechanisms that underlie heart failure and therapeutic interventions.

For this study, we investigated the effect of QL treatment for up to 8 weeks in rats with heart failure, and we compared its effect with the Western drug benazepril. We found that QL has a significant beneficial effect in the treatment of heart failure in terms of improving the levels of markers of heart failure. Compared to benazepril, QL provides unique therapeutic benefits with respect to regulating metabolites related to inflammation and cardiac hypertrophy in heart failure.

## Methods

### Animal model and study design

This study was approved by the ethics committee of the Integrated Traditional Chinese and Western Medicine Research Institute of Hebei Province, and all animal experiments were conducted in accordance with the “Guide for the Care and Use of Laboratory Animals” published by the National Institutes of Health (No. 85–23, revised 1996).

#### Rat model of heart failure after acute myocardial infarction

Male Sprague-Dawley rats weighing 200–220 g were purchased from Huafukang Biological Technology Co., Ltd. (Beijing, China) and housed in group cages at 22 °C with a 12 h light/12 h dark cycle for three days with ad libitum access to standard chow and water. The rats were randomly assigned to undergo either acute myocardial infarction (AMI) surgery or sham surgery. AMI surgical model was induced by ligation of the left anterior descending. Five weeks after ligation, echocardiography was performed, and rats with left ventricular ejection fraction ≤45% were diagnosed as having heart failure (n = 120 rats). The rats in the sham surgery group were treated exactly the same as the AMI group, except the artery was not ligated (n = 48 rats).

#### Interventions

Starting on day 1 following surgery, the rats were treated with QL (1 g/kg/day), benazepril (10 mg/kg/day), or physiological saline solution (PSS), administered by oral gavage. QL is the medicinal powder of Qiliqiangxin capsules, which was provided by Shijiazhuang Yiling Pharmaceutical (Shijiazhuang, Hebei, China). The material was produced by a verified process and contains consistent concentrations of herbal constituents^8^. Benazepril was used as a positive control for intervention and was purchased from Beijing Novartis Pharma Co., Ltd. (Beijing, China; batch number X2137). PSS was used as a negative control.

#### Animal groups and study design

**Figure 1:**
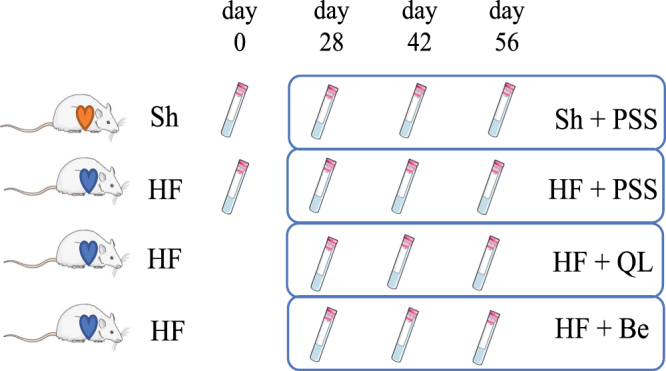
Study design. Be: Benazepril; HF: heart failure group; PSS: physiological saline solution; QL: medicinal powder of Qiliqiangxin capsules; Sh: Sham surgery group. Each tube represents a group. This figure was drawn by the author J. Fu using the software Adobe Illustrator (version CC 2015.2.0) and the image bank of Servier Medical Art. URL to the images are http://smart.servier.com/smart_image/rat/ and http://smart.servier.com/smart_image/tube-29/. Servier Medical Art by Servier is licensed under a Creative Commons Attribution 3.0 Unported License. https://creativecommons.org/licenses/by/3.0/.

The rats were assigned to their respective groups on day 0. The rats in the sham surgery group were randomized into the following four groups: one group was sacrificed immediately after randomization (day 0), and the other three groups received PSS and were sacrificed on day 28, day 42, or day 56 (see Fig. 1). The rats with heart failure were randomized into the following ten groups: one group was sacrificed after randomization; the other nine groups were divided into intervention type (QL, benazepril, or PSS) and sacrifice day (day 28, day 42, or day 56). On the indicated days, plasma samples were collected in lithium heparin tubes and immediately centrifuged at 1050 × *g* for 10 min at 4 °C; 500-µl aliquots were stored at −80 °C and used for the following measurements.

### Biochemical analysis

Biochemical parameters which were clinically relevant to heart failure were measured in all plasma samples. Angiopoietin-like 4 (AngPTL-4), neuregulin-1 (NRG-1), 3-nitrotyrosine (3-NT), arginine vasopressin (AVP), and calcitonin gene-related peptide (CGRP) were each measured using the respective ELISA. Renin (RI), angiotensin II (AII), aldosterone (ALD) were measured by using a radioimmunoassay kit, and free fatty acids (FFAs) were measured using an FFA kit.

### Metabolomics profiling

Plasma samples were measured using organic acids, oxidative stress, and oxylipins platforms in order to cover a wide spectrum in the metabolomics profile. From each sample, 50, 100, and 150 µl samples of plasma were prepared using the TECAN EVO Freeware (Männedorf, Swaziland). The stability of the platforms used was monitored using quality control (QC) samples and duplicate samples. A QC pool was made from study samples and used for QC and batch correction for all platforms. These QC samples were measured after every 10 samples. In addition, 15% of samples were run as duplicate samples for each platform (where sample volume allowed).

All analyses were performed by the Biomedical Metabolomics Facility Leiden at Leiden University using standard operating procedures based on previously published methods^13–15^. Details regarding these methods are provided in the Supplementary Methods, and the target metabolites are listed in Supplementary Tables S1-S3.

#### Organic acids

The organic acid platform uses untargeted gas chromatography-mass spectrometry combined with a target list of well-characterized metabolites for the analysis of organic acids, fatty acids, sugars, amines, and primary metabolites in complex biological matrices. The method uses a combination of an oximation reaction using methoxyamine hydrochloride and a silylation reaction using *N*-methyl-*N*-(trimethylsilyl)trifluoroacetamide^13^.

#### Oxylipins

The oxylipin platform uses a complete targeted liquid chromatography-tandem mass spectrometry approach with a defined target compound list covering the classic and non-classic eicosanoids derived from various polyunsaturated fatty acids (PUFAs), including *n*-6 and *n*-3 PUFAs such as linoleic acid, arachidonic acid, eicosapentaenoic acid, and docosahexaenoic acid^14^.

#### Oxidative stress‒related metabolites

The oxidative stress platform uses LC-MS/MS and covers various isoprostane classes together with their respective prostaglandin isomers derived from different PUFAs, including *n*-6 and *n*-3 PUFAs such as dihomo-γ-linoleic acid, arachidonic acid, and eicosapentaenoic acid. This platform also includes signaling lipids from the sphingosine and sphinganine classes, and their phosphorylated forms, as well as three classes of lysophosphatidic acids. The three lysophosphatidic acid classes include lysophosphatidic acids (LPAs), alkyl-lysophosphatidic acids (aLPAs), and cyclic-lysophosphatidic acids (cLPAs), all of which range from C14 to C22 chain length species^15^.

### Data preprocessing

The raw organic acid and oxylipin data were preprocessed using Agilent MassHunter Quantitative Analysis software (version B.05.01; Agilent Technologies, Santa Clara, CA). The oxylipin response was quantified using the peak area ratios between the target analyte and the respective internal standard. The raw oxidative stress data were evaluated using LabSolutions software (version 5.72; Shimadzu, Kyoto, Japan) by integrating the assigned multiple reaction monitoring peaks and normalizing each peak to the respective internal standard. Where available, a deuterated version of the target compound was used as an internal standard; otherwise, the closest-eluting internal standard was used.

For each platform, we first used performed internal standard correction followed by additional corrections using the QC samples in order to compensate for possible changes in the sensitivity of the mass spectrometer during the measurements. The quality of these corrections was evaluated by examining duplicate samples. Only metabolites with a relative standard deviation <0.30 in the duplicate samples and QC samples were used for subsequent analyses. Metabolites with a level below its limit of detection were replaced by 50% of the minimal value of the corresponding metabolite.

### Statistical analysis

Preprocessed metabolomics data were standardized prior to statistical analysis. Data were analyzed using an independent t-test, an one-way ANOVA, or a two-way ANOVA. All analyses were performed using IBM SPSS for Windows (version 24, Armonk, NY).

### Data availability

The datasets that were generated and analyzed in this study are available from the corresponding author upon request.

## Results and Discussion

### Time-dependent effects of QL on heart failure

#### QL improves biochemical parameters in a rat model of heart failure

At four time points (see Fig. 1), we measured nine well-known biochemical parameters relevant to heart failure in order to investigate the pathophysiological processes that underlie the disease. These nine biochemical parameters were: RI, AII, ALD, CGRP, AVP, FFAs, AngPTL-4, NRG-1, and 3-NT. RI, AII, and ALD play key roles in the renin-angiotensin-aldosterone system (RAAS), an important pathway involved in the pathogenesis of heart failure; in clinical practice, inhibiting the production of RI, AII and/or ALD can significantly increase survival in heart failure^16^. CGRP is a potent vasodilator, and increased CGRP levels in patients with appear to be correlated with disease severity^17^. AVP is an endogenous antidiuretic hormone, and increased levels can lead to an inappropriate retention of water in the body and constrict blood vessels in patients with heart failure^18^. FFAs play an important role in energy metabolism in the heart, and increased-FFA levels have been reported in patients with heart failure^19,20^. AngPTL-4 is a member of the AngPTL family of proteins and is involved in both lipoprotein metabolism and angiogenesis^21^. NRG-1 is a member of the epidermal growth factor family and has been reported to improve cardiac function and has beneficial effects in heart failure^22^. Finally, 3-NT is a product of tyrosine nitration, which is mediated by reactive nitrogen species, and increased levels of NT have been associated with systemic inflammation in patients with heart failure^23^.

**Figure 2:**
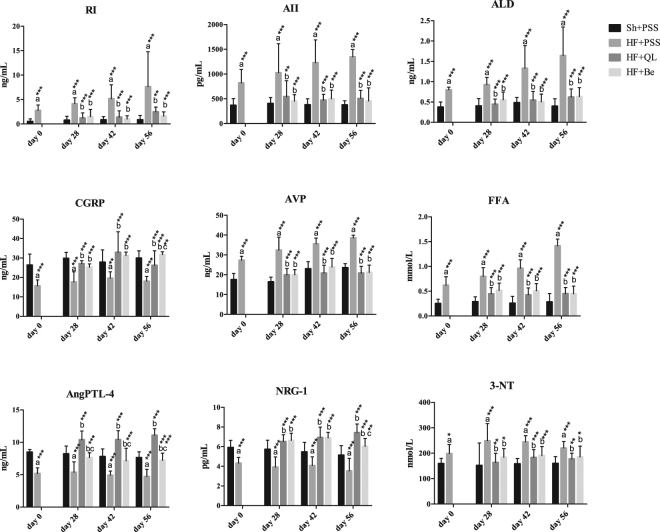
Biochemical parameters measured in the plasma of rats in the sham surgery/placebo-treated (Sh + PSS) and heart failure/placebo-treated (HF + PSS), heart failure/QL-treated (HF + QL), and heart failure/benazepril-treated (HF + Be) groups in day 0, day 28, day 42, and day 56. Values are presented as the mean ± SD. a: versus Sh + PSS; b: versus HF + PSS; c: versus HF + QL. *p < 0.05, **p < 0.01, ***p < 0.001.

Independent t-test and one-way ANOVA with LSD post hoc test were used to analyze the differences in biochemical parameters between the various surgery and treatment groups at all four time points (see Supplementary Table S4). As shown in Fig. 2, at all four time points, all nine biochemical parameters differed significantly between the placebo-treated heart failure (HF + PSS) and sham (Sh + PSS) groups. In the rats with heart failure, both interventions (i.e., QL and benazepril) significantly reversed all nine parameters compared to the HF + PSS group. Moreover, the effect of QL on AngPRL-4 was stronger than benazepril at all three time points, whereas CGRP and NRG-1 differed significantly between the two intervention groups only on day 56. Compared to the sham group, AngPLT-4 levels were higher in the HF + QL group, but not in the HF + Be group (see Supplementary Table S5). Thus, QL may have be more potent than benazepril in stimulating the production of AngPLT-4 in order to compensate for the damage caused by heart failure, resulting in higher levels than in the sham surgery group. This suggests that QL may have additional effects in terms of regulating AngPTL-4 related pathways in rats with heart failure, thus improving cardiac function by regulating lipoprotein metabolism and angiogenesis.

#### QL affects potential metabolic markers for heart failure

**Figure 3:**
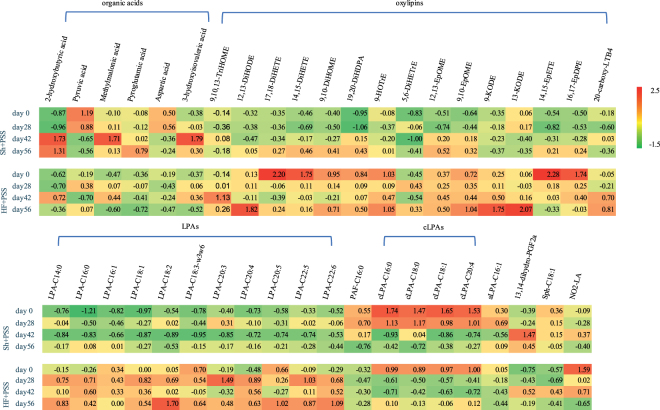
Summary of the 41 metabolites that differed significantly between the sham/placebo-treated group (Sh + PSS) and the heart failure/placebo-treated group (HF + PSS) at the indicated time points. Each number represents the mean standardized value (Z score) for each metabolite in the indicated group, and the color of each cell indicates the magnitude and direction of each Z score.

**Potential metabolic markers for heart failure:** In total, 147 metabolites were measured, including 25 organic acids, 71 oxylipins, and 51 oxidative stress metabolites. These data were first normalized by converting each measurement to a Z score using the formula [(X-$\bar{{\rm{X}}}$)/SD], where $X$ is the value obtained for a given metabolite in one sample, $\bar{{\rm{X}}}$ is the mean value of that metabolite in all samples, and SD is the standard deviation of the metabolite in all samples. Next, a two-way ANOVA was used to determine which metabolites differed significantly between the heart failure and sham rats. A total of 41 metabolites differed significantly between the HF + PSS and Sh + PSS groups and are shown in Fig. 3, which shows the mean Z scores for each metabolite in each group, with each cell colored to indicate the magnitude of the mean Z score.

A total of 6 organic acids, 4 cLPAs, aLPA (C16:1), platelet-activating factor (PAF C16:0), oxidative stress marker 13,14-dihydro-PGF_2a_, and sphingosine C18:1 were significantly lower in the HF + PSS group compared to the Sh + PSS group; in contrast, 15 oxylipins, 11 LPAs, and the nitrosative stress marker NO_2_-LA were significantly higher in the HF + PSS group. The metabolites 17,18-DiHETE, 19,20-DiHDPA, 5,6-DiHETrE, and 9,10-DiHOME have been reported to have a positive correlation with mortality among patients in systolic heart failure who received an implantable cardioverter defibrillator a primary prevention^24^. The enzymes and pathways involved in LPA metabolism and signaling play a role in atherosclerosis, as well as in experimental models of cardiovascular disease^25^. Sphingosine can be phosphorylated to produce sphingosine-1-phosphate, a potent signaling lipid involved in the pathogenesis of heart failure^24^. Nitrosative stress and oxidative stress have also been implicated in the pathogenesis of heart failure^26^. Importantly, our results provide the first evidence that downstream products of nitrosative and oxidative stress (NO_2_-LA and 13,14-dihydro-PGF_2a_) play a role in a rat model of heart failure. Our results also provide the first evidence that methylmalonic acid, pyroglutamic acid, aspartic acid, and 3-hydroxyisovaleric acid are associated with heart failure. The remaining metabolites identified in our study have been previously reported to be associated—either directly or indirectly—with heart failure^27–30^. Therefore, the metabolites identified in our analysis may play a key role in the pathogenesis of heart failure, and they may be useful as novel metabolic markers for disease progression in heart failure and/or targets for improving treatment outcome.

**The effects of QL and benazepril on potential metabolic markers:** Next, we examined the effects of treating heart failure rats with QL and benazepril with respect to these potential metabolic markers using a two-way ANOVA, with the intervention considered as one factor. Our analysis revealed that QL and benazepril significantly affected 21 and 16 metabolites (see Table 1), respectively; nine metabolites were affected by both interventions QL significant reduced 11 out of the 15 oxylipins, whereas benazepril significantly decreased only 5 oxylipins. In addition, QL significantly decreased all 11 LPAs, whereas benazepril decreased only 4 LPAs. The levels of each metabolite in each group are shown in Supplementary Fig. S1. Taken together, these results suggest that QL may be more effective than benazepril at regulating oxylipin and LPA metabolism in rats with heart failure.

**Table 1:** Summary of the effects of QL and benazepril on potential metabolic markers in rats with heart failure

Metabolite	*p*-value	
	HF + PSS vs. HF + QL	HF + PSS vs. HF + Be
Pyroglutamic acid	0.129	**0**.**004** ↑
Aspartic acid	0.140	**0**.**011** ↑
9,10,13-TriHOME	**0**.**045** ↓	0.087
12,13-DiHODE	**0**.**002** ↓	**0**.**009** ↓
9,10-DiHOME	**0**.**030** ↓	0.392
9-HOTrE	**0**.**002** ↓	**0**.**002** ↓
5,6-DiHETrE	**3**.**24E-05** ↓	0.277
12,13-EpOME	**0**.**011** ↓	0.073
9,10-EpOME	**4**.**34E-04** ↓	**2**.**408E-04** ↓
9-KODE	**2**.**02E-05** ↓	**0**.**001** ↓
13-KODE	**1**.**11E-07** ↓	**0**.**004** ↓
16,17-EpDPE	**0**.**027** ↓	0.226
LPA-C14:0	**0**.**006** ↓	0.107
LPA-C16:0	**0**.**040** ↓	0.081
LPA-C16:1	**0**.**013** ↓	0.711
LPA-C18:1	**0**.**003** ↓	0.106
LPA-C18:2	**4**.**45E-06** ↓	**7**.**141E-05** ↓
LPA-C18:3-ω3ω6	**0**.**017** ↓	0.441
LPA-C20:3	**0**.**001** ↓	0.081
LPA-C20:4	**0**.**007** ↓	**0**.**003** ↓
LPA-C20:5	**1**.**81E-04** ↓	0.812
LPA-C22:5	**2**.**26E-04** ↓	**0**.**005** ↓
LPA-C22:6	**0**.**003** ↓	**1**.**084E-04** ↓
PAF-C16:0	0.553	**0**.**001** ↑
cLPA-C16:0	0.391	**0**.**020** ↑
cLPA-C18:1	0.251	**0**.**036** ↑
aLPA-C16:1	0.216	**0**.**029** ↑
NO_2_-LA	0.839	**0**.**024** ↓

Notes: *p*-values shown in bold indicate *p* < 0.05; HF, heart failure; PSS, physiological saline solution

↓: Lower levels in the QL- or benazepril-treated groups (HF + QL or HF + Be) compared to the placebo-treated group (HF + PSS);

↑: Higher levels in the QL- or benazepril-treated groups (HF + QL or HF + Be) compared to the placebo-treated group (HF + PSS).

QL treatment had no significant effect on organic acid levels, whereas benazepril increased both pyroglutamic acid and aspartic acid. Aspartic acid is widely used to reduce cardiac ischemia/reperfusion during cardiac surgery^31^. Thus, the increased in aspartic acid may explain the potentially cardioprotective effect of benazepril. In other hand, high levels of aspartic acid may have toxic effects on the kidneys and salivary glands in rats^32^. Therefore, further study is needed in order to investigate more thoroughly the putative relationship between benazepril-induced changes in aspartic acid and cardioprotection.

### Regulation of heart failure‒related pathways by QL: a possible working mechanism

Studying a drug’s mechanism of action is one of the most important tasks in drug research. Because Qiliqiangxin Capsules showed positive clinical effects in the treatment of heart failure, we used a reverse pharmacology approach to investigate the underlying mechanism. Specifically, we used metabolomics profiling and biochemical assessment to explore the possible molecular targets of QL, using benazepril as a positive control.

#### Pathway analysis

**Figure 4:**
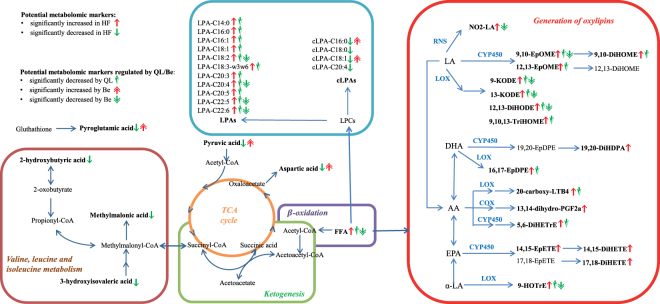
Pathway analysis of metabolites that are increased or decreased in rats with heart failure, and the effect of treatment with QL or benazepril. AA: arachidonic acid; Be: benazepril; COX: cyclooxygenase; CYP450: cytochrome P450; DHA: docosahexaenoic acid; EPA: eicosapentaenoic acid; FFA: free fatty acid; HF: heart failure; LA: linoleic acid; LOX: lipoxygenase; RNS: reactive nitrogen species; TCA: tricarboxylic acid.

To investigate the working mechanism of QL, the related biochemical parameters and metabolites are summarized in Fig. 4 using pathways reported in the KEGG (Kyoto Encyclopedia of Genes and Genomes) database, the published literature, and the Boehringer metabolic chart^33^.

Heart failure, a complex condition involving pathological perturbation in a variety cardiac metabolic systems, has been linked to inflammation^23^. Oxylipins are potent inflammatory markers that have been associated with responses to cardiovascular disease, host defense, tissue injury, and surgical intervention^14^. *In vivo*, oxylipins are present as oxygenated 20-carbon lipids derived from PUFAs via enzymatic (e.g., COX, CYP450, or LOX) or non-enzymatic oxidative or nitrosative (e.g., RNS) reactions (see Fig. 4.). Here, we found that rats with heart failure had significantly higher levels of 15 oxylipins compared to rats in the sham surgery group, indicating that heart failure evokes an inflammatory response. As discussed above (see section ‘The effects of QL and benazepril on potential metabolic markers’), treating rats in the heart failure group with QL reduced 11 of these 15 oxylipins, compared to only five oxylipins reduced by benazepril treatment. These results indicate that QL is more effective than benazepril at modulating inflammation due to heart failure.

In the untreated rats with heart failure (i.e., the HF + PSS group), 11 LPAs were significantly increased, and 4 cLPAs were significantly decreased. LPAs are growth factor‒like phospholipid mediators, and one of the major pathways for their production is via the hydrolysis of lysophosphatidylcholine (LPC) by lysophospholipase D^34^. Cyclic analogs of LPAs (cLPAs) are also generated from lysophosphatidylcholine by an enzyme that shares several peptide fragments with lysophospholipase D^27^. Therefore, LPAs and cLPAs are produced from lysophosphatidylcholines via the activity of two similar phospholipases. QL treatment significantly decreased all LPAs; in contrast, benazepril treatment reduced only four LPAs and increased two cLPAs. These results suggest that both QL and benazepril exert effects on the LPA/cLPA-related pathways. Thus, QL may have a stronger effect in terms of inhibiting LPA generation, whereas benazepril may only modestly inhibit LPA generation while stimulating the production cLPAs. LPAs have important functions in extracellular signaling, including inducing platelet aggregation and vascular smooth muscle cell contraction, increasing arterial blood pressure, and promoting angiogenesis in rats^25,34,35^. Angiogenesis is closely correlated with cardiac hypertrophy, which is an adaptive response to physiological and pathological overload^36^. Initially, this response is functional, compensatory, and adaptive; however, when sustained, the response can lead to structural changes that become self-perpetuating and pathogenic^37^. In contrast, cLPAs have unique functions such as inhibiting LPA-induced platelet aggregation^38^. Therefore, QL may improve cardiac hypertrophy by decreasing LPA levels, thereby contributing to recovery from heart failure over the long term.

In the healthy mammalian heart, the majority of energy is produced from the β-oxidation of fatty acids; in heart failure, oxidation of myocardial fatty acids is reduced^10,39^. This finding may partially explain our finding of increased levels of FFAs in rats with heart failure. Importantly, FFA levels were significantly decreased in rats that were treated with QL or benazepril, suggesting that both interventions increase myocardial fatty acid oxidation in order to normalize FFA levels in heart failure. In placebo-treated rats with heart failure (i.e. HF + PSS group), several organic acid levels were decreased compared to rats in the sham surgery group (i.e. Sh + PSS group), which may indicate the changes in the tricarboxylic acid (TCA) cycle, as well as in the valine, leucine, and isoleucine degradation pathways. Valine, leucine, and isoleucine are branched-chain amino acids (BCAAs)^33^ and BCAAs may provide an alternative source of energy for maintaining heart function^40^. Thus, decreased levels of organic acids in rats with heart failure may suggest a perturbation in energy production in this group. In our study, QL had no significant effect terms of modulating these organic acids, whereas benazepril significantly increase two organic acids related to the TCA cycle (pyruvic acid and aspartic acid).

#### Comparison between QL and benazepril treatment in rats with heart failure

In this study, we used benazepril and PSS as a positive and negative control, respectively, for intervention in rats with heart failure. Benazepril is an ACE inhibitor that is used to treat heart failure by acting on the RAAS, thereby driving sodium and water retention and lowering blood pressure.

Both QL and benazepril significantly improved a number of disease-related biochemical parameters in rats with heart failure, including RI, AII, ALD, AVP, 3-NT, FFA, and CGRP, suggesting that their therapeutic effects are mediated by inhibiting RAAS, reducing water retention, decreasing reactive nitrogen species (RNS)-induced inflammation, and promoting the oxidation of myocardial fatty acids. Only three biochemical parameters—namely, AngPTL-4, CGRP and NRG-1—were affected differently between QL and benazepril treatment. In particular, AngPTL-4 levels were consistently higher in the QL-treated group compared with the benazepril-treated group (*p* < 0.001). AngPTL-4 is an inhibitor of lipoprotein lipase; thus increased levels of AngPTL-4 can reduce the production of lipoprotein-derived fatty acids, thereby stimulating lipolysis and fatty acid oxidation^41^. This finding suggests that QL may be more effective than benazepril at restoring lipid metabolism in heart failure. With respect to metabolomics pathways, QL was more effective than benazepril at controlling LPA and oxylipin levels. This suggests that QL may have unique therapeutic benefits with respect to controlling cardiac hypertrophy and inflammation associated with heart failure, thereby improving long-term outcome.

Benazepril is relatively safe and effective for treating patients with heart failure^42^. Although Qiliqiangxin Capsule is derived from a traditional Chinese medicine formula and has been used successfully in clinical practice, it is a relatively new medicine compared to benazepril. Therefore, the above-mentioned effects of QL on biochemical and metabolic markers may contribute to our understanding of its functional mechanisms in treating heart failure.

## Conclusions

In this study, we used targeted metabolomics profiling and biochemical assessment in plasma samples obtained from rats with heart failure treated under various conditions. In rats with heart failure, nine biochemical parameters and 41 metabolites differed significantly compared to rats in the sham surgery group. All of the biochemical parameters and metabolites identified in our study can be measured in plasma samples obtained from both humans and rats, and these markers should be examined further in studies involving patients with heart failure. QL significantly improved several clinically relevant biochemical parameters. Compared to benazepril, QL led to significantly higher levels of AngPLT-4, as well as lower levels of oxylipins and LPAs. These results suggest that QL may be therapeutically beneficial in terms of controlling cardiac hypertrophy and inflammation, as well as promoting lipid metabolism in heart failure, thereby providing a novel mechanism for promoting recovery in these patients.

## Electronic supplementary material

Supplementary information
