# Supplementary material for: A metabolomics study of Qiliqiangxin in a rat model of heart failure: a reverse pharmacology approach
Source: Sci Rep. 2018 Feb 27;8:3688. doi: 10.1038/s41598-018-22074-6 (PMC5829193; doi:10.1038/s41598-018-22074-6)
Supplement: Supplementary file 1 — Supplementary information [file 41598_2018_22074_MOESM1_ESM.pdf]

A metabolomics study of Qiliqiangxin in a rat model of heart failure: a reverse pharmacology approach

Junzeng Fu[1#], Liping Chang[2, 3#], Amy C. Harms[1, 4], Zhenhua Jia[3], Hongtao Wang[5]，Cong Wei[6]，Li Qiao[6], Shuyan Tian[2,3],Thomas Hankemeier[1, 4], Yiling Wu[2,3*] Mei Wang[7,8*]

[1] Department of Analytical Biosciences, Leiden Academic Center for Drug Research, Leiden University, Einsteinweg 55, 2333 CC, Leiden, The Netherlands
[2] Hebei Medical University, Shijiazhuang, Hebei 050017, P.R. China
[3] Yiling Hospital of Hebei Medical University, The Key Laboratory of State Administration of Traditional Chinese Medicine, Shijiazhuang, Hebei 050091, P.R. China
[4] Netherlands Metabolomics Centre, Leiden University, Einsteinweg 55, 2333 CC, Leiden, The Netherlands
[5] National Key Laboratory of Collateral Disease Research and Innovative Chinese Medicine, Shijiazhuang, Hebei 050035, P.R. China
[6] Hebei Key Laboratory of Collateral Disease, Shijiazhuang, Hebei 050035, P.R. China,
[7] LU-European Center for Chinese Medicine and Natural Compounds, Institute of Biology, Leiden University, Sylviusweg72, 2333BE, Leiden, The Netherlands
[8]SU BioMedicine, Sylviusweg72, 2333BE, Leiden, The Netherlands

[#] These authors equally contributed to the manuscript
[*] Co-corresponding authors

Correspondence to:
Professor Yiling Wu, Yiling Pharmaceutical Research Institute, 238 Tianshan Street, Shijiazhuang, Hebei 050035, P.R. China; E-mail: changliping08@163.com
Dr. Mei Wang, LU-European Center for Chinese Medicine and Natural Compounds, Institute of Biology, Leiden University, Sylviusweg72, 2333BE, Leiden, The Netherlands; E-mail: M.Wang@biology.leidenuniv.nl

Methods: metabolomic profiling

1 Organic acids profiling [1]

Sample preparation was done by doing first protein precipitation of 50 uL of sample with a crash solvent (methanol/H_2O) with ISTD added. After centrifugation and transferring the supernatant, the solvent was evaporated to complete dryness on the speedvac. Then, two-step derivatization procedures with oximation using methoxyamine hydrochloride as first reaction and silylation using MSTFA as second reaction were carried out. After this final step, the samples were transferred to the auto sampler vials and 1 μL was injected on GC-MS.

The metabolites were measured by gas chromatography on an Agilent Technologies 7890A equipped with an Agilent Technologies mass selective detector (MSD 5975C) and MultiPurpose Sampler (MPS, MXY016-02A, GERSTEL). Chromatographic separations were performed on a HP-5MS UI (5% Phenyl Methyl Silox), 30 m × 0.25 m ID column with a film thickness of 25 m, using helium as the carrier gas at a flow rate of 1.7 mL/min. A single-quadrupole mass spectrometer with electron impact ionization (EI, 70 eV) was used. The mass spectrometer was operated in SCAN mode mass range 50-500. The data could be re-analyzed to extract additional targets.

2 Oxylipins profiling [2]

The samples were spiked with antioxidant and internal standard mix. Then the samples were diluted with wash solution and transferred into a SPE cartridge. The SPE cartridge is a hydrophilic-lipophilic balance (HLB) (Oasis, Waters). Oxylipins were eluted with methanol and ethyl acetate. To concentrate, the eluate was gently dried under nitrogen stream and reconstituted in 50 μL of injection solvent (acetonitrile and methanol,1:1 v/v). In total 5 μL was injected per analysis.

The HPLC was coupled to electrospray ionization on a triple quadrupole mass spectrometer (Agilent 6490, San Jose, CA, USA). Separation was done by HPLC (Agilent 1290 infinity, San Jose, CA, USA) using an Ascentis® Express column (2.1x150 mm, 2.7 μm particles; Supelco, Bellefonte, PA, USA) with 0.35 mL flow during 28 min gradient. Oxylipins were detected in negative ion mode using dynamic Multiple Reaction Monitoring (MRM).

3 Oxidative stress metabolites profiling [3]

Each plasma sample was spiked with antioxidant and internal standard solutions. The extraction of the oxidative stress compounds is performed via LLE. To extract the analytes from the aqueous phase, butanol and ethylacetate are used. After collection, the organic phase is concentrated by first drying and then reconstitution in a smaller volume. After reconstitution, the extract is divided in two vials (one for each chromatography) and used for injection.

A Shimadzu system formed by three high pressure pumps (LC-30AD), a controller (CBM-20Alite), and autosampler (SIL-30AC) and an oven (CTO-30A) from Shimadzu Benelux, was coupled online with a LCMS-8050 Triple quadrupole mass spectrometer (Shimadzu) operated using LabSolutions data acquisition software (Version 5.72, Shimadzu). The

samples were analyzed by UPLC-MS/MS using a Kromasil Eternity XT C18 column (Akzo Nobel) for high pH and an Acquity BEH C18 column (Waters) for the low pH method. The Triple Quadrupole MS was used in polarity switching mode and all analytes were monitored in dynamic Multiple Reaction Monitoring (dMRM). Sphingosines C17:1 and C18:1, Sphinganines C17:0 and C18:0, PAF C16:0 and PAF C16:0-d4 were measured in positive ion mode. The other metabolites were detected in negative mode.

References:

1. Koek, M. M. *et al*. Semi-automated non-target processing in GC × GC-MS metabolomics analysis: applicability for biomedical studies. *Metabolomics* **7,** 1–14 (2011).
2. Strassburg, K. *et al*. Quantitative profiling of oxylipins through comprehensive LC-MS/MS analysis: application in cardiac surgery. *Anal. Bioanal. Chem.* **404,** 1413–26 (2012).
3. Fu, J. *et al*. Metabolomics profiling of the free and total oxidised lipids in urine by LC-MS/MS: application in patients with rheumatoid arthritis. *Anal. Bioanal. Chem.* (2016). doi:10.1007/s00216-016-9742-2

Tables

Table S1 List of detected metabolites in GC/MS platform

Metabolite	Formula	HMDB	InChI Key
FA C14:0 (Myristic acid)	C14H28O2	HMDB0000806	TUNFSRHWOTWDNC-UHFFFAOYSA-N
FA C18:0 (Stearic acid)	C18H36O2	HMDB0000827	QIQXTHQIDYTFRH-UHFFFAOYSA-N
FA C18:2	C18H32O2		
FA C18:3-ω3 (α-Linolenic acid)	C18H30O2	HMDB0001388	DTOSIQBPPRVQHS-PDBXOOCHSA-N
FA C 20:4-ω6 (Arachidonic acid)	C20H32O2	HMDB0001043	YZXBAPSDXZZRGB-DOFZRALJSA-N
2-hydroxybutyric acid	C4H8O3	HMDB0000008	AFENDNXGAFYKQO-UHFFFAOYSA-N
Citric acid	C6H8O7	HMDB0000094	KRKNYBCHXYNGOX-UHFFFAOYSA-N
Glutamic Acid	C9H9NO4	HMDB0000148	WHUUTDBJXJRKMK-VKHMYHEASA-N
Glutaric acid	C5H8O4	HMDB0000661	JFCQEDHGNNZCLN-UHFFFAOYSA-N
Glycolic acid	C2H4O3	HMDB0000115	AEMRFAOFKBGASW-UHFFFAOYSA-N
Malic acid	C4H6O5	HMDB0000744	BJEPYKJPYRNKOW-UHFFFAOYSA-N
2-ketoglutaric acid	C5H6O5	HMDB0000208	KPGXRSRHYNQIFN-UHFFFAOYSA-N
Succinic acid	C4H6O4	HMDB0000254	KDYFGRWQOYBRFD-UHFFFAOYSA-N
Fumaric acid	C4H4O4	HMDB0000134	VZCYOOQTPOCHFL-OWOJBTEDSA-N
Pyruvic acid	C3H4O3	HMDB0000243	LCTONWCANYUPML-UHFFFAOYSA-N
Methylmalonic acid	C4H6O4	HMDB0000202	ZIYVHBGGAOATLY-UHFFFAOYSA-N
Pyroglutamic acid	C5H7NO3	HMDB0000267	ODHCTXKNWHHXJC-VKHMYHEASA-N
Isocitrate	C6H8O7	HMDB0000193	ODBLHEXUDAPZAU-UHFFFAOYSA-N
3-Hydroxybutyric acid	C4H8O3	HMDB0000357	WHBMMWSBFZVSSR-UHFFFAOYSA-N
Aspartic acid	C4H7NO4	HMDB0000191	CKLJMWTZIZZHCS-REOHCLBHSA-N
3-Hydroxyisobutyric acid	C4H8O3	HMDB0000023	DBXBTMSZEOQQDU-UHFFFAOYSA-N
3-Hydroxyisovaleric acid	C5H10O3	HMDB0000754	AXFYFNCPONWUHW-UHFFFAOYSA-N
Glyceric acid	C3H6O4	HMDB0000139	RBNPOMFGQQGHHO-UHFFFAOYSA-N
Uracil	C4H4N2O2	HMDB0000300	ISAKRJDGNUQOIC-UHFFFAOYSA-N
3-Hydroxypropionic acid	C3H6O3	HMDB0000700	ALRHLSYJTWAHJZ-UHFFFAOYSA-N

Table S2. List of detected metabolites in oxylipin platform.

Metabolite	Formal name	Formula	Lipidmaps	InchI-Key
10-HDoHE	(+/-)-10-hydroxy-4Z,7Z,11E,13Z,16Z,19Z-docosahexaenoic acid	C22H32O3	LMFA04000027	DDCYKEYDTGCKAS-SKSHMZPZSA-N
11,12-DiHETrE	(±)11,12-dihydroxy-5Z,8Z,14Z-eicosatrienoic acid	C20H34O4	LMFA03050008	LRPPQRCHCPFBPE-LZXKBWHHSA-N
11,12-EpETrE	(±)11(12)-epoxy-5Z,8Z,14Z-eicosatrienoic acid	C20H32O3	LMFA03080014	DXOYQVHGIODESM-LZXKBWHHSA-N
11beta-13,14-dihydro-15-keto-PGF2a	9S,11R-dihydroxy-15-oxo-prost-5Z-en-1-oic acid	C20H34O5	LMFA03010203	VKTIONYPMSCHQI-KGILNJEGSA-N
11-HETE	11R-hydroxy-5Z,8Z,12E,14Z-eicosatetraenoic acid	C20H32O3	LMFA03060028	GCZRCCHPLVMMJE-WXMXURGXSA-N
12,13-DiHODE	(+/-)-12,13-dihydroxy-9Z,15Z-octadecadienoic acid	C18H32O4	LMFA02000046	RGRKFKRAFZJQMS-OOHFSOINSA-N
12,13-DiHOME	12,13-dihydroxy-9Z-octadecenoic acid	C18H34O4	LMFA01050351	CQSLTKIXAJTQGA-GJGKEFFFSA-N
12,13-EpOME	(+/-)-12(13)-epoxy-9Z-octadecenoic acid	C18H32O3	LMFA02000038	CCPPLLJZDQAOHD-FLIBITNWSA-N
12-HETE	12-hydroxy-5Z,8Z,10E,14Z-eicosatetraenoic acid	C20H32O3	LMFA03060088	ZNHVWPKMFKADKW-VXBMJZGYSA-N
12S-HEPE	12S-hydroxy-5Z,8Z,10E,14Z,17Z-eicosapentaenoic acid	C20H30O3	LMFA03070008	MCRJLMXYVFDXLS-UOLHMMFFSA-N
12S-HHTrE	12S-hydroxy-5Z,8E,10E-heptadecatrienoic acid	C17H28O3	LMFA03050002	KUKJHGXXZWHSBG-WBGSEQOASA-N
13,14-dihydro-15-keto-PGD2	9S-hydroxy-11,15-dioxo-prost-5Z-en-1-oic acid	C20H32O5	LMFA03010022	VSRXYLYXIXYEST-KZTWKYQFSA-N
13-HDoHE	(+/-)-13-hydroxy-4Z,7Z,10Z,14E,16Z,19Z-docosahexaenoic acid	C22H32O3	LMFA04000029	SEVOKGDVLLIUMT-SKSHMZPZSA-N
13-HODE	13S-hydroxy-9Z,11E-octadecadienoic acid	C18H32O3	LMFA02000154	HNICUWMFWZBIFP-BSZOFBHHSA-N
13-KODE	13-keto-9Z,11E-octadecadienoic acid	C18H30O3	LMFA02000016	JHXAZBBVQSRKJR-BSZOFBHHSA-N
14,15-DiHETrE	14,15-dihydroxy-5Z,8Z,11Z-eicosatrienoic acid	C20H34O4	LMFA03050010	SYAWGTIVOGUZMM-ILYOTBPNSA-N
14,15-DiHETrE	(±)14,15-dihydroxy-5Z,8Z,11Z-eicosatrienoic acid	C20H34O4	LMFA03050010	SYAWGTIVOGUZMM-KZTFMOQPSA-N
14,15-EpETE	(±)14,15-epoxy-5Z,8Z,11Z,17Z-eicosatetraenoic acid	C20H30O3	LMFA03000003	RGZIXZYRGZWDMI-IXQKDQKQSA-N
14,15-EpETrE	(±)14(15)-epoxy-5Z,8Z,11Z-eicosatrienoic acid	C20H32O3	LMFA03080013	JBSCUHKPLGKXKH-KZTFMOQPSA-N
14-HDoHE	(+/-)-14-hydroxy-4Z,7Z,10Z,12E,16Z,19Z-docosahexaenoic acid	C22H32O3	LMFA04000030	ZNEBXONKCYFJAF-BGKMTWLOSA-N
15-deoxy-delta-12,14-PGD2	9S-hydroxy-11-oxo-prosta-5Z,12E,14E-trien-1-oic acid	C20H30O4	LMFA03010051	QUGBPWLPAUHDTI-PLGLXCLHSA-N
15-HETE	15S-hydroxy-5Z,8Z,11Z,13E-eicosatetraenoic acid	C20H32O3	LMFA03060001	JSFATNQSLKRBCI-VAEKSGALSA-N
15-KETE	PE(18:0/20:4(5Z,8Z,11Z,13E)(15Ke))	$C_{43}H_{76}NO_9P$	LMGP20010004	YGJTUEISKATQSM-USWFWKISSA-N
15S-HEPE	15S-hydroxy-5Z,8Z,11Z,13E,17Z-eicosapentaenoic acid	C20H30O3	LMFA03070009	WLKCSMCLEKGITB-DBVSHIMFSA-N
15S-HETrE	15S-hydroxy-8Z,11Z,13E-eicosatrienoic acid	C20H34O3	LMFA03050007	IUKXMNDGTWTNTP-OAHXIXLCSA-N
16,17-EpDPE	(±)16(17)-epoxy-4Z,7Z,10Z,13Z,19Z-docosapentaenoic acid	C22H32O3	LMFA04000037	BCTXZWCPBLWCRV-QCAYAECISA-N
16-HDoHE	(±)16-hydroxy-4Z,7Z,10Z,13Z,17E,19Z-docosahexaenoic acid	C22H32O3	LMFA04000031	CSXQXWHAGLIFIH-VUARBJEWSA-N
17,18-DiHETE	(+/-)-17,18-dihydroxy-5Z,8Z,11Z,14Z-eicosatetraenoic acid	C20H32O4	LMFA03060078	XYDVGNAQQFWZEF-JPURVOHMSA-N
17-HDoHE	(±)17-hydroxy-4Z,7Z,10Z,13Z,15E,19Z-docosahexaenoic acid	C22H32O3	LMFA04000032	SWTYBBUBEPPYCX-VIIQGJSXSA-N
19,20-DiHDPA	(±)19,20-dihydroxy-4Z,7Z,10Z,13Z,16Z-docosapentaenoic acid	C22H34O4	LMFA04000043	FFXKPSNQCPNORO-MBYQGORISA-N
1a,1b-dihomo-PGF2a	(+/-)-19(20)-epoxy-4Z,7Z,10Z,13Z,16Z-docosapentaenoic acid	C22H32O3	LMFA04000038	OSXOPUBJJDUAOJ-MBYQGORISA-N
20-carboxy-LTB4	5S,12R-dihydroxy-6Z,8E,10E,14Z-eicosatetraene-1,20-dioic acid	C20H30O6	LMFA03020016	SXWGPVJGNOLNHT-VFLUTPEKSA-N
20-HDoHE	(+/-)-20-hydroxy-4Z,7Z,10Z,13Z,16Z,18E-docosahexaenoic acid	C22H32O3	LMFA04000033	YUZXOJOCNGKDNI-LFVREGEGSA-N
20-HETE	20-hydroxy-5Z,8Z,11Z,14Z-eicosatetraenoic acid	C20H32O3	LMFA03060009	NNDIXBJHNLFJJP-DTLRTWKJSA-N
5,6-DiHETrE	5S,6S-dihydroxy-7E,9E,11Z,14Z-eicosatetraenoic acid	C20H32O4	LMFA03060018	UVZBUUTTYHTDRR-WAQVJNLQSA-N
5,6-EpETrE	(±)5(6)-epoxy-8Z,11Z,14Z-eicosatrienoic acid	C20H32O3	LMFA03080017	VBQNSZQZRAGRIX-GSKBNKFLSA-N
5-HETE	5S-hydroxy-6E,8Z,11Z,14Z-eicosatetraenoic acid	C20H32O3	LMFA03060002	KGIJOOYOSFUGPC-JGKLHWIESA-N
5-iPF2a-VI	5,9S,11R-trihydroxy-(8S)-prosta-6E,14Z-dien-1-oic acid	C20H34O5	LMFA03110011	RZCPXIZGLPAGEV-SUHLLOIRSA-N
5-KETE	5-oxo-6E,8Z,11Z,14Z-eicosatetraenoic acid	C20H30O3	LMFA03060011	MEASLHGILYBXFO-XTDASVJISA-N

5S,6S-DiHETE	5S,6S-dihydroxy-7E,9E,11Z,14Z-eicosatetraenoic acid	C20H32O4	LMFA03060018	UVZBUUTTYHTDRR-WAQVJNLQSA-N
5S,6S-Lipoxin A4	5S,6R,15S-trihydroxy-7E,9E,11Z,13E-eicosatetraenoic acid	C20H32O5	LMFA03040001	IXAQOQZEOGMIQS-SSQFXEBMSA-N
5S-HEPE	5S-hydroxy-6E,8Z,11Z,14Z,17Z-eicosapentaenoic acid	C20H30O3	LMFA03070010	FTAGQROYQYQRHF-GHWNLOBHSA-N
5S-HpETE	5S-hydroperoxy-6E,8Z,11Z,14Z-eicosatetraenoi	C20H32O4	LMFA03060012	JNUUNUQHXIOFDA-XTDASVJISA-N
6-keto-PGF1a	6-oxo-9S,11R,15S-trihydroxy-prost-13E-en-1-oic acid	C20H34O6	LMFA03010001	KFGOFTHODYBSGM-ZUNNJUQCSA-N
6-trans-LTB4	5S,12R-dihydroxy-6E,8E,10E,14Z-eicosatetraenoic acid	C20H32O4	LMFA03020013	VNYSSYRCGWBHLG-UKNWISKWSA-N
8,9-DiHETrE	8,9-dihydroxy-5Z,11Z,14Z-eicosatrienoic acid	C20H34O4	LMFA03050006	DCJBINATHQHPKO-TYAUOURKSA-N
8,9-EpETrE	(±)8(9)-epoxy-5Z,11Z,14Z-eicosatrienoic acid	C20H32O3	LMFA03080019	DBWQSCSXHFNTMO-ZZMPYBMWSA-N
8-HETE	(±)8-hydroxy-5Z,9E,11Z,14Z-eicosatetraenoic acid	C20H32O3	LMFA03060086	NLUNAYAEIJYXRB-HEJOTXCHSA-N
8-HETrE	8S-hydroxy-9E,11Z,14Z-eicosatrienoic acid	C20H34O3	LMFA03050011	SKIQVURLERJJCK-RDCCVJQZSA-N
8S,15S-DiHETE	8S,15S-dihydroxy-5Z,9E,11Z,13E-eicosatetraenoic acid	C20H32O4	LMFA03060050	NNPWRKSGORGTIM-HCCKYKKOSA-N
9,10,13-TriHOME	9,10,13-Trihydroxy-11-octadecenoic acid	C18H34O5	LMFA02000168	NTVFQBIHLSPEGQ-BUHFOSPRSA-N
9,10-DiHOME	9,10-dihydroxy-12Z-octadecenoic acid	C18H34O4	LMFA02000229	XEBKSQSGNGRGDW-CJWPDFJNSA-N
9,10-EpOME	(+/-)-9(10)-epoxy-12Z-octadecenoic acid	C18H32O3	LMFA02000037	FBUKMFOXMZRGRB-XKJZPFPASA-N
9,12,13-TriHOME	9S,12S,13S-trihydroxy-10E-octadecenoic acid	C18H34O5	LMFA02000014	MDIUMSLCYIJBQC-MVFSOIOZSA-N
9-HEPE	(±)-9-hydroxy-5Z,7E,11Z,14Z,17Z-eicosapentaenoic acid	C20H30O3	LMFA03070029	OXOPDAZWPWFJEW-IMCWFPBLSA-N
9-HODE	(±)-9-hydroxy-10E,12Z-octadecadienoic acid	C18H32O3	LMFA02000151	NPDSHTNEKLQQIJ-ZJHFMPGASA-N
9-HOTrE	9S-hydroxy-10E,12Z,15Z-octadecatrienoic acid	C18H30O3	LMFA02000024	RIGGEAZDTKMXSI-MEBVTJQTSA-N
9-KODE	9-oxo-10E,12Z-octadecadienoic acid	C18H30O3	LMFA02000274	LUZSWWYKKLTDHU-ZJHFMPGASA-N
HepoxilinA3	8-hydroxy-11S,12S-epoxy-5Z,14Z,9E-eicosatrienoic acid	C20H32O4	LMFA03090005	SGTUOBURCVMACZ-SEVPPISGSA-N
PGA2	9-oxo-15S-hydroxy-5Z,10Z,13E-prostatrienoic acid	C20H30O4	LMFA03010035	MYHXHCUNDDAEOZ-FOSBLDSVSA-N
PGD2	9S,15S-dihydroxy-11-oxo-5Z,13E-prostadienoic acid	C20H32O5	LMFA03010004	BHMBVRSPMRCCGG-OUTUXVNYSA-N
PGE1	9-oxo-11R,15S-dihydroxy-13E-prostaenoic acid	C20H34O5	LMFA03010134	GMVPRGQOIOIIMI-DWKJAMRDSA-N
PGE2	9-oxo-11R,15S-dihydroxy-5Z,13E-prostadienoic acid	C20H32O5	LMFA03010003	XEYBRNLFEZDVAW-ARSRFYASSA-N
PGE3	9-oxo-11R,15S-dihydroxy-5Z,13E,17Z-prostatrienoic acid	C20H30O5	LMFA03010135	CBOMORHDRONZRN-QLOYDKTKSA-N
PGF1a	9S,11R,15S-trihydroxy-13E-prostaenoic acid	C20H36O5	LMFA03010137	DZUXGQBLFALXCR-CDIPTNKSSA-N
PGF2a	9S,11R,15S-trihydroxy-5Z,13E-prostadienoic acid	C20H34O5	LMFA03010002	PXGPLTODNUVGFL-YNNPMVKQSA-N
PGJ2	11-oxo-15S-hydroxy-prosta-5Z,9,13E-trien-1-oic acid	C20H30O4	LMFA03010019	UQOQENZZLBSFKO-POPPZSFYSA-N
TXB1	9S,11,15S-trihydroxy-thrombox-13Eenoic acid	C20H36O6	LMFA03030008	JSDWWNLTJCCSAV-VZBVYBAISA-N
TXB2	9S,11,15S-trihydroxy-thromboxa-5Z,13E-dien-1-oic acid	C20H34O6	LMFA03030002	XNRNNGPBEPRNAR-JQBLCGNGSA-N
TXB3	9S,11,15S-trihydroxy-thromboxa-5Z,13E,17Z-trien-1-oic acid	C20H32O6	LMFA03030006	OYPPJMLKAYYWHH-NXJDUNGTSA-N
Δ12-PGJ2	11-oxo-15S-hydroxy-5Z,9Z,13E-prostatrienoic acid	C20H30O4	LMFA03010020	TUXFWOHFPFBNEJ-GJGHEGAFSA-N

Table S3 List of detected metabolites in oxidative stress platform.

Metabolite	Formal name	Formula	Lipid Maps ID	InchI-Key
13,14-dihydro-PGF2a	9S,11R,15S-trihydroxy-5Z-prostenoic acid	C20H36O5	LMFA03010079	
5-iPF2a-VI	5,9S,11R-trihydroxy-6E,14Z-prostadienoic acid-cyclo[8S,12R]	C20H34O5	LMFA03110010	
5-iPF2a-VI (353-115) f2 a	5,9S,11R-trihydroxy-6E,14Z-prostadienoic acid-cyclo[8S,12R]	C20H34O5	LMFA03110011	
8,12-iPF2a-VI	12-iso-5(R),6E,14Z-Prostaglandin F2α	C20H34O5		RZCPXIZGLPAGEV-DCOIXEBESA-N
8-iso-15(R)-PGF2a	9α,11α,15R-trihydroxy-(8β)-prosta-5Z,13E-dien-1-oic acid	C20H34O5		PXGPLTODNUVGFL-PGWUFSIFSA-N
8-iso-PGA1	9-oxo-15S-hydroxy-10Z,13E-prostadienoic acid-cyclo[8S,12S]	C20H32O4	LMFA03110008	
8-iso-PGA2	9-oxo-15S-hydroxy-(8β)-prosta-5Z,10,13E-trien-1-oic acid	C20H30O4		MYHXHCUNDDAEOZ-UKUWKSPLSA-N
8-iso-PGE1	9-oxo-11α,15S-dihydroxy-(8β)-prost-13E-en-1-oic acid	C20H34O5	LMFA03110002	GMVPRGQOIOIIMI-JCPCGATGSA-N
8-iso-PGE2	9-oxo-11α,15S-dihydroxy-(8β)-prosta-5Z,13E-dien-1-oic acid	C20H32O5	LMFA03110003	XEYBRNLFEZDVAW-CLQOMRTCSA-N
8-iso-PGF2a	9α,11α,15S-trihydroxy-(8β)-prosta-5Z,13E-dien-1-oic acid	C20H34O5	LMFA03110001	PXGPLTODNUVGFL-NAPLMKITSA-N
aLPA-C16:1				
aLPA-C18:1				
cLPA-C16:0	1-hexadecanoyl-sn-glycero-2,3-cyclic-phosphate	C19H37O6P	LMGP00000057	WLYRJURLXSFXRK-GOSISDBHSA-N
cLPA-C18:0	1-octadecanoyl-sn-glycero-2,3-cyclic phosphate	C21H41O6P	LMGP00000055	BAAJXXGEGGUMMX-HXUWFJFHSA-N
cLPA-C18:1	1-(9Z-octadecenoyl)-sn-glycero-2,3-cyclic phosphate	C21H39O6P	LMGP00000056	ZUUIRLHAEZAVCP-GDCKJWNLSA-N
LPA-C14:0	1-tetradecanoyl-sn-glycero-3-phosphate	C17H35O7P	LMGP10050007	FAZBDRGXCKPVJU-MRXNPFEDSA-N
LPA-C16:0	1-hexadecanoyl-sn-glycero-3-phosphate	C19H39O7P	LMGP10050006	YNDYKPRNFWPPFU-GOSISDBHSA-N
LPA-C16:1	1-(9Z-hexadecenoyl)-glycero-3-phosphate	C19H37O7P	LMGP10050016	GLGQZYWTNAOWHT-JTHGQSKGSA-N
LPA-C18:0	1-octadecanoyl-sn-glycero-3-phosphate	C21H43O7P	LMGP10050005	LAYXSTYJRSVXIH-HXUWFJFHSA-N
LPA-C18:1	1-(9Z-octadecenoyl)-sn-glycero-3-phosphate	C21H41O7P	LMGP10050008	WRGQSWVCFNIUNZ-GDCKJWNLSA-N
LPA-C18:2	1-(9Z,12Z-octadecadienoyl)-glycero-3-phosphate	C21H39O7P	LMGP10050017	ZQTAMPRZFOOEEP-KKFOGOCZSA-N
LPA-C18:3-w3w6	1-(6Z,9Z,12Z-octadecatrienoyl)-glycero-3-phosphate	C21H37O7P	LMGP10050023	KWFUSJZUJLGPTO-ZJVPVJIZSA-N
LPA-C20:1	1-(11Z-eicosenoyl)-glycero-3-phosphate	C23H45O7P	LMGP10050026	OAYFHKCHTMNTLM-MZMPXXGTSA-N
LPA-C20:3	1-(8Z,11Z,14Z-eicosatrienoyl)-glycero-3-phosphate	C23H41O7P	LMGP10050028	LZSKMXWVALYCLG-CSLWLMPESA-N
LPA-C20:4	1-(5Z,8Z,11Z,14Z-eicosatetraenoyl)-sn-glycero-3-phosphate	C23H39O7P	LMGP10050013	XBFQFMCUPHZKTI-NZRYSPDRSA-N
LPA-C20:5	1-(5Z,8Z,11Z,14Z,17Z-eicosapentaenoyl)-glycero-3-phosphate	C23H37O7P	LMGP10050033	DKFJPTBTSHHMCQ-MWRKSYAASA-N
LPA-C22:4	1-(7Z,10Z,13Z,16Z-docosatetraenoyl)-glycero-3-phosphate	C25H43O7P	LMGP10050020	KSDVXYAHZXLGDS-XSQXPFHXSA-N
LPA-C22:5				
LPA-C22:6	1-(4Z,7Z,10Z,13Z,16Z,19Z-docosahexaenoyl)-glycero-3-phosphate	C25H39O7P	LMGP10050019	UWHSPTWBPTXYMF-DOYOFOADSA-N
NO2-LA	10-nitro,9Z,12Z-octadecadienoic acid	C18H31NO4	LMFA01120001/2	LELVHAQTWXTCLY-XYWKCAQWSA-N
PAF-C16:0	1-hexadecyl-2-acetyl-sn-glycero-3-phosphocholine	C26H54NO7P	LMGP01020046	HVAUUPRFYPCOCA-AREMUKBSSA-N
PGA1	9-oxo-15S-hydroxy-10Z,13E-prostadienoic acid	C20H32O4	LMFA03010005	BGKHCLZFGPIKKU-LDDQNKHRSA-N
PGA2	9-oxo-15S-hydroxy-5Z,10Z,13E-prostatrienoic acid	C20H30O4	LMFA03010035	MYHXHCUNDDAEOZ-FOSBLDSVSA-N
PGD2	9S,15S-dihydroxy-11-oxo-5Z,13E-prostadienoic acid	C20H32O5	LMFA03010004	BHMBVRSPMRCCGG-OUTUXVNYSA-N
PGE1	9-oxo-11R,15S-dihydroxy-13E-prostaenoic acid	C20H34O5	LMFA03010134	GMVPRGQOIOIIMI-DWKJAMRDSA-N
PGE2	9-oxo-11R,15S-dihydroxy-5Z,13E-prostadienoic acid	C20H32O5	LMFA03010003	XEYBRNLFEZDVAW-ARSRFYASSA-N
PGE3	9-oxo-11R,15S-dihydroxy-5Z,13E,17Z-prostatrienoic acid	C20H30O5	LMFA03010135	CBOMORHDRONZRN-QLOYDKTKSA-N
PGF1a	9S,11R,15S-trihydroxy-13E-prostaenoic acid	C20H36O5	LMFA03010137	DZUXGQBLFALXCR-CDIPTNKSSA-N
PGF2a	9S,11R,15S-trihydroxy-5Z,13E-prostadienoic acid	C20H34O5	LMFA03010002	PXGPLTODNUVGFL-YNNPMVKQSA-N

S-1-P-C18:1	Sphing-4-enine-1-phosphate	C18H38NO5P	LMSP01050001	DUYSYHSSBDVJSM-KRWOKUGFSA-N
Spha-1-P-C18:0	Sphing-4-enine	C18H37NO2	LMSP01010001	WWUZIQQURGPMPG-KRWOKUGFSA-N
Spha-C18:0	Sphinganine	C18H39NO2	LMSP01020001	OTKJDMGTUTTYMP-ZWKOTPCHSA-N
Sph-C18:1	Sphinganine-1-phosphate	C18H40NO5P	LMSP01050002	YHEDRJPUIRMZMP-ZWKOTPCHSA-N
Unknown 333-271 [a]				
Unknown 353-183 [a]				
cLPA-C18:2				
cLPA-C20:3				
cLPA-C20:4				
F3-series 351 [a]				
LPA_3_455 [b]				
LPA_483 [b]				

a: Four extra metabolites were found in some samples during the low pH chromatography and they were putatively annotated as:5-iPF2α VI (353-115) f2, F3-series 351, unknown 333-271 and unknown 353-183. 5-iPF2α VI (353-115) f2 and F3-series 351 have a characteristic fragment for 5-iPF2α VI and PGF3a respectively.
b: Two extra metabolites were found in the samples for the high pH chromatography and they were putatively annotated as: LPA_483, LPA_3_455. Those compounds have the same characteristic fragment as the LPA family and they were named with the m/z value of their parent ion.

Table S4. The differences (p values) of biochemical parameters between/ among groups at each time point.

Biochemical parameter	T0 [a]	T1 [b]				T2 [b]				T3 [b]			
	HF+PSS *v.s* Sh+PSS	HF+PSS *v.s* Sh+PSS	HF+QL *v.s.*HF+PSS	HF+Be *v.s.* *HF+PSS*	HF+Be *v.s.*HF+QL	HF+PSS *v.s* Sh+PSS	HF+QL *v.s.*HF+PSS	HF+Be *v.s.* *HF+PSS*	HF+Be *v.s.*HF+QL	HF+PSS *v.s* Sh+PSS	HF+QL *v.s.*HF+PSS	HF+Be *v.s.* *HF+PSS*	HF+Be *v.s.*HF+QL
AI	<0.001	<0.001	<0.001	<0.001	0.625	<0.001	<0.001	<0.001	0.648	<0.001	0.003	<0.001	0.584
AII	<0.001	<0.001	0.002	<0.001	0.528	<0.001	<0.001	<0.001	0.856	<0.001	<0.001	<0.001	0.516
ALD	<0.001	<0.001	<0.001	<0.001	0.143	<0.001	<0.001	<0.001	0.834	<0.001	<0.001	<0.001	0.961
CGRP	<0.001	<0.001	<0.001	<0.001	0.176	0.003	<0.001	<0.001	0.604	<0.001	<0.001	<0.001	0.006
AVP	<0.001	<0.001	<0.001	<0.001	0.987	<0.001	<0.001	<0.001	0.130	<0.001	<0.001	<0.001	0.812
FFA	<0.001	<0.001	<0.001	<0.001	0.223	<0.001	<0.001	<0.001	0.276	<0.001	<0.001	<0.001	0.964
AngPLT-4	<0.001	<0.001	<0.001	<0.001	<0.001	<0.001	<0.001	<0.001	<0.001	<0.001	<0.001	<0.001	<0.001
NRG-1	<0.001	<0.001	<0.001	<0.001	0.734	<0.001	<0.001	<0.001	0.935	<0.001	<0.001	<0.001	0.003
3-NT	0.018	<0.001	0.002	0.014	0.386	<0.001	<0.001	<0.001	0.583	<0.001	0.003	0.010	0.584

a: The p values were calculated by independent student t-test (two-way);
b: The p values were calculated by one-way ANOVA with LSD posthoc test.

Table S5 The comparisons between heart failure groups and sham surgery groups at each time point.

Biochemical parameter	Group v.s. Sh+PSS	T1		T2		T3	
		Mean difference with Sh+ PSS	p value	Mean difference with Sh+ PSS	p value	Mean difference with Sh+ PSS	p value
GCRP	HF+PSS	12.149	<0.001	8.249	0.003	11.844	<0.001
	HF+Be	4.529	0.002	-3.408	0.221	-1.803	0.328
	HF+QL	2.774	0.044	-5.072	0.099	3.643	0.058
AngPLT-4	HF+PSS	2.833	<0.001	2.886	<0.001	2.905	<0.001
	HF+Be	0.591	0.271	0.649	0.270	0.405	0.355
	HF+QL	-2.191	<0.001	-2.628	<0.001	-3.471	<0.001
NRG-1	HF+PSS	1.803	<0.001	1.395	0.001	1.606	0.001
	HF+Be	-0.900	0.017	-1.417	0.001	-0.901	0.040
	HF+QL	-0.782	0.036	-1.455	0.002	-2.291	<0.001

Mean difference with Sh+ PSS=Mean $_{\text{Sh+PSS}}$ – Mean $_{\text{group listed on second column}}$

p values were calculated by by one-way ANOVA with LSD posthoc test.

Figure

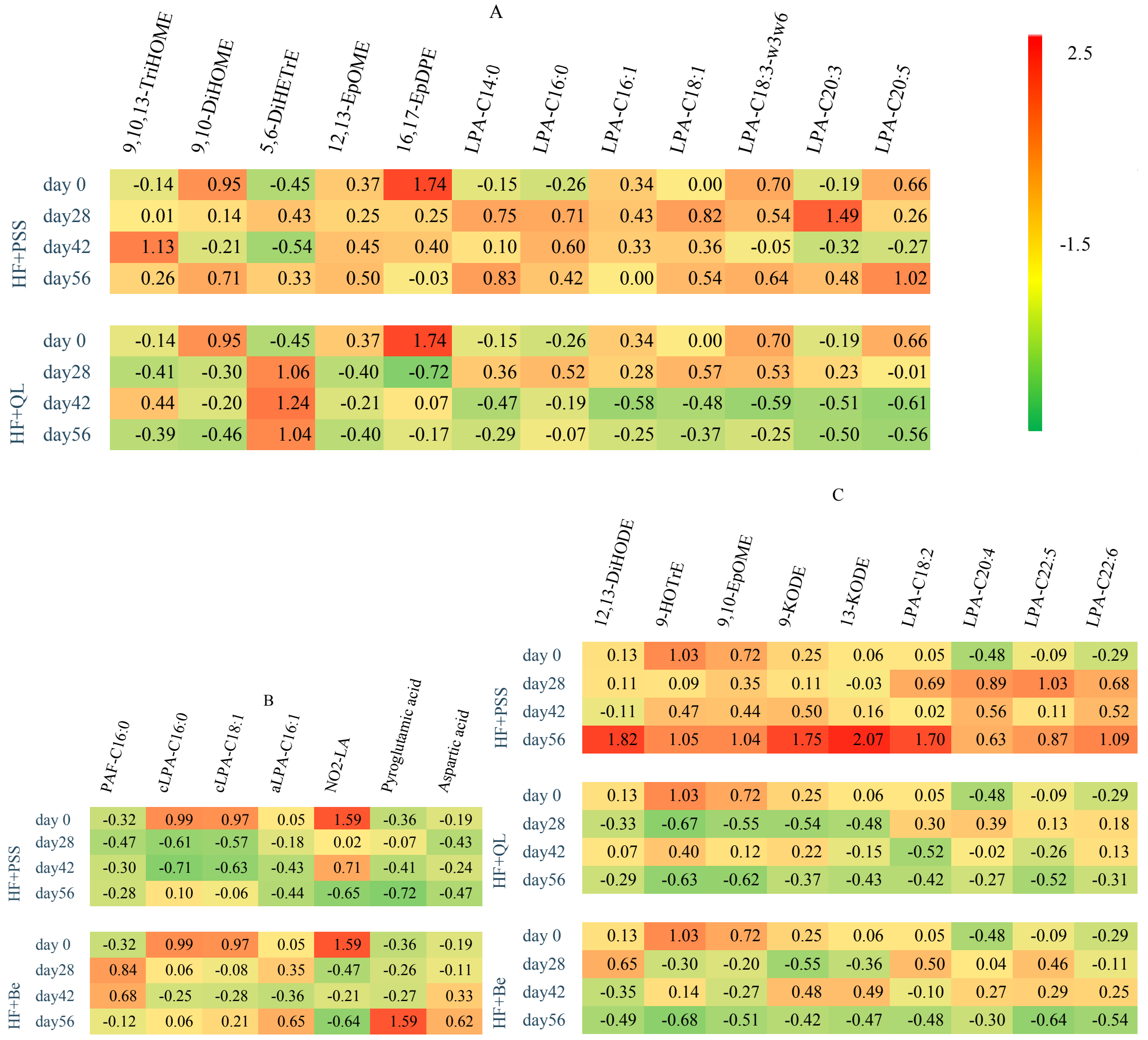

Fig S1. The effects of QL and benazepril interventions on the potential metabolomic markers by by (A) QL intervention only (HF+PPS v.s. HF+QL, $p<0.05$); (B) by Be intervention only (HF+PPS v.s. HF+Be, $p<0.05$); (C) by both interventions (HF+PPS v.s. HF+QL, $p<0.05$ and HF+PPS v.s. HF+Be, $p<0.05$).
The numbers represent the mean of standardized metabolite values (Z scores) in each group; red color indicates a high mean value and the green color indicates a low value.
